# The effectiveness and applicability of mindfulness intervention in psychological adaptation after sports injury: a systematic review

**DOI:** 10.1080/00049530.2024.2357627

**Published:** 2024-05-26

**Authors:** Siqi Liu, Young-Eun Noh

**Affiliations:** Faculty of Sports and Exercise Science, Universiti Malaya, Kuala Lumpur, Malaysia

**Keywords:** Athlete, psychological rehabilitation, mindfulness, injury recovery, intervention protocol

## Abstract

**Objectives:**

This systematic review aimed to explore the role of mindfulness interventions (MI) in the psychological adaptation of athletes following injuries.

**Methods:**

Adhering to the Preferred Reporting Items for Systematic Reviews and Meta-Analyses (PRISMA) guidelines, this study conducted a systematic review of the effects of MI on the psychological adaptation of injured athletes.

**Results:**

The synthesized evidence indicates that MI has diverse positive effects on the psychological adaptation of injured athletes. These effects include reduced anxiety and depression, increased pain tolerance, elevated mindfulness levels, improved acceptance levels, and enhanced psychological well-being. Additionally, this study discusses the application of MI, considering factors such as dosage, timing, methods (online vs. offline, group vs. individual), implementers, and types of intervention. It also identifies four limitations: unverified efficacy, restricted applicability to specific injuries, minimal impact on physiological recovery, and potential adverse effects.

**Conclusions:**

This research provides valuable insights into the positive impact of MI on the psychological adaptation of injured athletes. It emphasizes the necessity for standardized protocols and highlights research gaps in optimal dosage, timing, and potential adverse effects.

## KEY POINTS

What is already known about this topic:

(1) Mindfulness practice can contribute to the holistic well-being of athletes.

(2) Mindfulness interventions aid in the psychological adaptation of injured individuals.

(3) Mindfulness interventions are commonly used as complementary and alternative practices in the recovery process of injured athletes.

What this topic adds:

(1) This review finds that mindfulness interventions positively affect the psychological adaptation of injured athletes by alleviating symptoms related to anxiety and depression, increasing pain tolerance, mindfulness levels, acceptance levels, and overall psychological well-being.

(2) This review identifies five key aspects to consider when implementing mindfulness interventions to enhance the psychological adaptation of injured athletes (i.e. dosage, timing, methods, implementers, and types of intervention).

(3) Four limitations of existing mindfulness intervention methods were identified in implementing mindfulness interventions for injured athletes (i.e. unverified efficacy, limited applicability to specific types of sports injuries, minimal impact on physiological recovery outcomes, and potential adverse effects).

## Introduction

Athletes are susceptible to various psychological challenges after sports injuries (Gervis et al., 2023; Haugen, 2022). Injured athletes face challenges such as fear of re-injury, lack of confidence, depression, and stress disorders (Gervis et al., 2020, 2023; Seguin & Culver, 2022; Yang et al., 2022). These psychological obstacles can negatively influence their surgical outcomes, recovery timelines, and post-return sports performance and pose a heightened risk of subsequent sports injuries (Barber-Westin & Noyes, 2020; Daley et al., 2021; Rogers et al., 2023).

The Biopsychosocial Model of Sports Injury Rehabilitation suggests that psychological interventions – such as biofeedback, goal setting, imagery/relaxation, self-talk, and combinations thereof – can influence athletes’ cognitive behaviours, thereby promoting their psychological adaptation to negative emotions following an injury (Brewer et al., 2002). This effectiveness stems from the requirement that athletes learn new skills or behaviours to effectively cope with both the physical and psychological aspects of the rehabilitation process (Brewer et al., 2002). In sports, psychological adaptation refers to athletes’ continually evolving ability to respond effectively to stressors, such as injuries, and take appropriate actions (Tenenbaum et al., 2015). Psychological adaptation covers a range of psychological constructs such as acceptance, mindfulness skills, psychological flexibility, the acceptance of negative emotions, and reductions in anxiety and depression. These constructs are key components in how individuals adjust to changes, challenges, or stressors in their lives. They are particularly relevant in contexts such as recovering from illness, coping with stress, or adjusting to significant life changes, where adaptive psychological responses can significantly affect outcomes. In the context of this paper on sports injury, psychological adaptation specifically refers to the psychological adjustments athletes make in response to an injury event.

Mindfulness, an ancient Buddhist practice, is defined as “paying attention in a particular way: on purpose, in the present moment, and non-judgmentally” (Kabat-Zinn, 2023, p. 28). This expansive framework encompasses various practice types, such as contemplation, chanting, visualization, and body scans (Matko et al., 2021). These practices have gained considerable traction in sports research and practical applications (Gardner & Moore, 2020). Research to date has demonstrated that mindfulness practice can reduce injury risk among athletes, ease physical discomfort, enhance sleep hygiene, aid in recovery from post-training fatigue, and improve sports performance (Herrero et al., 2021; Holguin-Ramirez et al., 2020; Josefsson et al., 2021; Mihajlovic et al., 2023; Moreton et al., 2022; Reiche et al., 2023; Scrivin et al., 2022; Zadeh et al., 2019). Additionally, mindfulness training has positively influenced athletes’ mental well-being by decreasing sports-related anxiety and mental fatigue, boosting resilience, and regulating impulsiveness levels (Brown et al., 2020; Coimbra et al., 2021; Liu et al., 2021; Myall et al., 2023; Naderi et al., 2020; Tingaz et al., 2022). Furthermore, there is considerable evidence that mindfulness practice supports psychological adaptation in athletes across various competition stages, including pre-game, in-game, and post-game scenarios (Coimbra et al., 2021; Gao & Zhang, 2023; Josefsson et al., 2021; Naderi et al., 2020).

Mindfulness interventions (MI) demonstrate superior effectiveness compared to specific physical interventions in the psychological rehabilitation of athletes post-injury (Shirvani et al., 2021). Additionally, MI has been acknowledged by American sports medicine experts as a supplementary and alternative approach to facilitate the rehabilitation process for injured athletes (Kent et al., 2020). Consequently, the consensus statement by the American College of Sports Medicine (2017) states that psychologists frequently incorporate MI into the psychological rehabilitation of athletes following injuries. Given the capability of mindfulness practice to regulate psychological adaptation, psychologists have integrated MI into the rehabilitation processes of injured or ill individuals to support their psychological adaptation (Wielgosz et al., 2019). For example, some researchers have used MI to enhance patient treatment adherence and acceptance of injuries and reduce anxiety and depression through approaches such as Mindfulness-Based Stress Reduction (MBSR) and Mindfulness and Acceptance-Based Intervention (MABI) (Clement et al., 2020; Finlay et al., 2021; Forbes & Johnson, 2022; Kwok et al., 2023; Lee et al., 2022; Lundgren et al., 2022; Matthews & Wang, 2022; Wells et al., 2019). Moreover, researchers have integrated MI with other therapeutic modalities to collectively promote patients’ psychological adaptation, incorporating mindfulness into Cognitive Behavioural Therapy (CBT), also known as Mindfulness-Based Cognitive Therapy (MBCT), and Acceptance Commitment Therapy (ACT) to alleviate patients’ subjective fatigue, depression, and perceived pain (Bredero et al., 2023; Herbert et al., 2022; Interian et al., 2023).

Despite the known benefits of MI on athletes’ mental health broadly (Myall et al., 2023), there remains a specific and unaddressed gap concerning how MI impacts the psychological adaptation of athletes during the critical period of rehabilitation post-injury. This gap is highlighted by the absence of a comprehensive synthesis of evidence particularly focused on the rehabilitation phase, as noted by Gledhill and Forsdyke (2021) and Noetel et al. (2019). Given the potential positive impact of MI on psychological adaptation both during competitions and in the aftermath of injuries or illnesses, this study aims to conduct a thorough review of articles on MI and its relation to the psychological adaptation of injured athletes. Specifically, this systematic review seeks to answer the following key question: What impact does MI have on the psychological adaptation of athletes after sports injuries?

## Methodology

This study adhered to the Preferred Reporting Items for Systematic Reviews and Meta-Analyses (PRISMA) guidelines (Moher, 2009). To broaden the scope of retrieval, we employed a mixed retrieval strategy, which included both electronic database searching and hand searching. The electronic database search was conducted using a Boolean search strategy, developed under the guidance of a librarian. The databases included in our electronic search were Web of Science, Psychology & Behavioral Sciences Collection, PubMed, SPORTDiscus, and Scopus. Our search criteria encompassed titles, abstracts, and keywords. The keywords for various types of mindfulness were derived from existing literature, specifically from a comprehensive overview of mindfulness techniques (Matko et al., 2021). The searching keywords include: (mindfulness* OR meditat* OR mantra* OR yoga* OR “MBSR” OR “MBCT” OR “Vipassana” OR “satipaṭṭhāna*” OR “anapanasati*” OR “Zen*” OR “Pranayama*” OR “Sudarshan*” OR “Kriya*” OR zazen* OR “Shambhala*” OR buddhis* OR tai*chi* OR qi*gong* OR “self compassion*” OR “body scan*” OR contemplation* OR chant* OR visualiz*) AND (athlet* OR runner* OR sport* OR player* OR competition*) AND (injur* OR rehabilitat* OR recover* OR treatment* OR therap* OR trauma* OR fracture* OR overuse* OR surg* OR “return to sport*” OR “RTS” OR “return to play*” OR “RTP” OR “return to compet*” OR “RTC”). The last retrieval was on 31 January 2024.

Hand searching included both forward and backward citation tracking. In addition to all studies identified through the PRISMA guidelines, we also conducted reference mining from previous systematic reviews related to the role of mindfulness in athletes. These reviews included works by Brooks et al. (2022), Brown et al. (2020); Furie et al. (2023); Gennarelli et al. (2020); Mihajlovic et al. (2023); Myall et al. (2023); Noetel et al. (2019); and Yang et al. (2022).

The inclusion criteria for this study comprised four points: 1) The studies included must target participants who are injured athletes (encompassing both chronic and acute injuries) or athletes preparing to return to sports after an injury; 2) The interventions conducted in the included studies must be related to mindfulness (including both adjunctive therapies and standalone interventions); 3) Articles from all publication years are included; 4) Articles must be published in English-language journals.

The exclusion criteria for this study included eight points: 1) interventions or strategies unrelated to mindfulness; 2) topics not related to psychological adaptation after sports injury; 3) articles with English titles and abstracts but with non-English main bodies; 4) grey literature (e.g., preprints, book chapters, theses, conference papers, or organizational reports); 5) articles not accessible in full text; 6) secular mindfulness practices, such as prayer because practices based on specific religions lack universal applicability in clinical settings; 7) review articles, as they only summarize previous research findings and propose research directions without generating new research conclusions; 8) articles that only involve mindfulness self-report questionnaire surveys without actual MI.

After saving all eligible articles from the mixed searching process and relevant systematic reviews in Zotero software, we conducted the literature screening process. This process consisted of three steps: 1) duplicate removal, 2) primary screening, and 3) secondary screening. Duplicate removal was conducted using Zotero. During the primary screening, two authors independently evaluated the titles and abstracts of all articles saved in Zotero, based on the inclusion and exclusion criteria. Articles that met the initial criteria were saved in Zotero with their full texts. The secondary screening involved an independent examination of these full-text articles identified during the primary screening. Studies that met any of the exclusion criteria were excluded from Zotero, with clear explanations provided in the PRISMA diagram. Any discrepancies arising during the primary and secondary screening processes were resolved through discussions among all authors.

The literature screening process was followed by two authors independently extracting, analysing, and coding data from the included full-text papers. The extracted data included various elements such as authors, publication year, study objectives, and results (see Table 1).

**Table 1.** The summary of findings from included articles (chronological order).

Author(s)	Year	Aim	Results
Mahoney and Hanrahan	2011	To examine the effect of an adapted ACT intervention in addressing individuals’ adherence to rehabilitation protocols and their general psychological well-being	The adapted ACT intervention contributed to accepting negative emotions such as depression, boredom, and anxiety. The adapted ACT intervention facilitated adherence to rehabilitation behaviours.
Bennett and Lindsay	2016	To investigate the impact of ACT intervention on the psychological challenges faced by athletes when returning to sports after injury	The ACT intervention alleviated perceived pain. The ACT intervention reduced anxiety during returning to sports. The ACT intervention improved sleep quality.
Mohammed et al.	2018	To investigate whether MBSR intervention could reduce injured athletes’ pain perception, enhance their mindfulness levels, and boost positive emotions during the rehabilitation process	The MBSR intervention improved pain tolerance and mindfulness levels.
Shortway et al.	2018	To design an MI protocol named the Return to ACTion for injured athletes.	The Return to ACTion protocol cultivated mindfulness levels, acceptance levels, and psychological flexibility. The Return to ACTion protocol contributed to rehabilitation compliance behaviours.
Moesch et al.	2020	To examine whether the MABI can enhance injured athletes’ mindfulness level, acceptance level, and psychological well-being	The MABI contributed to injured athletes’ mindfulness levels, acceptance levels, and psychological well-being.
Moesch	2023	To design an MABI to facilitate the psychological adaptation of injured athletes	The MABI contributed to the recovery of athletes from an acute injury.

ACT = acceptance and commitment therapy; MI = mindfulness intervention; MBSR = mindfulness-based stress reduction; MABI = mindfulness and acceptance-based intervention.

Additionally, for articles related to experimental interventions, two authors independently summarized and categorized details of the experimental design, including intervention type, timing, modality, dosage, and implementer. They also compiled information on participant specifics, such as the number of participants, gender distribution, types of sports involved, age demographics, and the nature of injuries. These comprehensive details are presented in Table 2. The discrepancies in the summarized results (including both Tables 1 and 2) were collaboratively discussed by all authors of this study until a consensus was reached.

**Table 2.** The summary of experimental design from included experimental studies (chronological order).

Author(s) and Year	Intervention				Participants				
	Type	Timing	Modality and Dosage	Implementer	Number	Gender	Sports	Age	Injuries
Mahoney and Hanrahan (2011)	Adapted ACT	1 to 4 months before the return to sports	Offline (Not provided)	Professionals who had received relevant meditation course training	*N* = 4	Male (*n* = 2), Female (*n* = 2)	Skiing athletes, football players, and sailing athletes	18 to 49	ACL
Mohammed et al. (2018)	MBSR	3–6 months post-injury	Online (720 minutes) and offline (Not provided)	Not provided	*N* = 20	Male (*n* = 14), Female (*n* = 6)	College athletes (i.e., tennis, kickboxing, bodybuilding, running, football, basketball, and cyclist)	21 to 36	ACL, CL, and TS
Moesch et al. (2020)	MABI	1 to 6 months post-injury	Online (960 minutes) and offline (240 minutes)	Sports psychologists with extensive experience in meditation intervention	*N* = 6	Male (*n* = 2), Female (*n* = 4)	Elite and sub-elite athletes	18 to 39	ACL, CL, MEN, CAR, PT, and AT

*Note*. ACT = acceptance and commitment therapy; MBSR = mindfulness-based stress reduction; MABI = mindfulness and acceptance-based interventions; ACL = anterior cruciate ligament; CL = collateral ligament; MEN = injury of meniscus; CAR = injury of cartilage; PT = injury of patella tendon; AT = rupture of Achilles tendon; TS = peroneal tendon subluxation.

The methodological quality appraisal utilized the Mixed Methods Appraisal Tool (MMAT; Hong et al., 2018). The MMAT addresses five prevalent study designs within mixed studies reviews, with each design having five criteria to assess the content validity of the empirical articles. There are five fundamental criteria for evaluating the methodological quality of each study design. Each criterion is rated on a scale of “yes”, “no”, and “cannot tell”. Additionally, the MMAT includes two screening questions. If the answer to either screening question is “no” or “cannot tell”, it may suggest that the paper is not an empirical study, thus rendering it ineligible for further methodological quality assessment using the MMAT.

## Results

**Figure 1.**
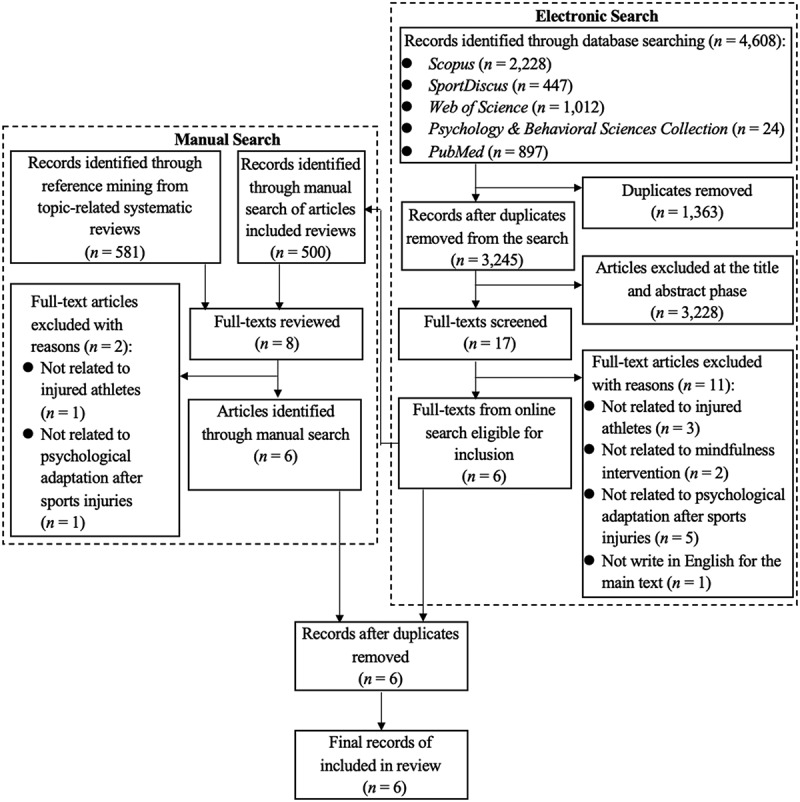
The PRISMA flow diagram.

An electronic search and hand search yielded a total of 4,608 and 1,081 articles respectively (see Figure 1). Following the three-step literature screening process, only six articles were included in this review. These articles included three empirical experimental studies (Mahoney & Hanrahan, 2011; Moesch et al., 2020; Mohammed et al., 2018), one experimental protocol design and feasibility validation for MI (Shortway et al., 2018), one case study (Bennett & Lindsay, 2016), and one non-empirical study that designed an MI protocol (Moesch, 2023).

The non-empirical study did not pass the screening questions of MMAT and therefore did not undergo further methodological quality assessment. Among the remaining five articles, three are non-randomized quantitative studies (Mahoney & Hanrahan, 2011; Moesch et al., 2020; Mohammed et al., 2018) and two are qualitative studies (Bennett & Lindsay, 2016; Shortway et al., 2018). According to the MMAT evaluation, the study by Shortway et al. (2018) lacked practical validation of the intervention’s effectiveness, thus reducing the credibility of the study results. The other four studies met all five criteria for content validity in the methodological quality assessment (see Table 3).

**Table 3.** MMAT quality appraisal of studies (chronological order).

Author(s)	Screening questions		Qualitative					Quantitative (non-randomized)					Comments
Mahoney and Hanrahan (2011)	✓	✓						✓	✓	✓	✓	✓	
Bennett and Lindsay (2016)	✓	✓	✓	✓	✓	✓	✓						
Shortway et al. (2018)	✓	✓	✓	✓	X	X	–						The effectiveness of the experimental intervention protocol awaits practical validation.
Mohammed et al. (2018)	✓	✓						✓	✓	✓	✓	✓	
Moesch et al. (2020)	✓	✓						✓	✓	✓	✓	✓	
Moesch (2023)	✓	X											This is a non-empirical study.

“✓” means that the criterion is met, “X” indicates that the criterion is not met, and “–” means that there is not enough information in the paper to judge if the criterion is met or not. Additionally, the assessment columns for three study designs (i.e., quantitative randomized controlled trials, mixed methods, and quantitative descriptive), have been removed because these types of study designs were not utilized in the articles included in this review.

The five studies that passed the methodological quality assessment, along with the non-empirical study, collectively demonstrated that MI contributed to the psychological adaptation of injured athletes. These positive psychological adaptations included mitigating symptoms related to decreased anxiety and depression, as well as increasing pain tolerance, mindfulness levels, acceptance levels, and psychological well-being (Bennett & Lindsay, 2016; Mahoney & Hanrahan, 2011; Moesch et al., 2020; Mohammed et al., 2018). Additionally, MI was found to influence adherence to rehabilitation behaviours (Bennett & Lindsay, 2016; Mahoney & Hanrahan, 2011), which was also a contributing factor to psychological adaptation following sports injuries (Coronado et al., 2020). Next, we provide a detailed review of these six articles.

Bennett and Lindsay (2016) documented a case study involving a 6-month psychological intervention for a female hockey player recovering from a back injury. This player was a member of the U21 women’s field hockey team and had completed physical therapy post-injury. Despite assurances from her physiotherapist of a full physical recovery, she still harboured apprehensions about returning to sport and the potential for re-injury, leading to avoidance behaviours such as frequent absences from training and team-building sessions. These anxieties manifested in heightened anxiety levels and sleep difficulties. Employing the framework of ACT, Bennett and Lindsay implemented a structured intervention plan over 6 months, including 12 face-to-face sessions lasting 60–90 minutes each. The primary objective was to bolster the athlete’s capacity for acceptance, enhance her mindfulness skills, and foster psychological flexibility. MI was integrated into pre-training warm-up stretches and pre-sleep relaxation routines to facilitate pain management and address sleep hygiene issues. After the MI, she experienced a significant reduction in anxiety levels and sleep disturbances. Furthermore, there was a notable decrease in fear-driven avoidance behaviours, accompanied by an enhancement in sports confidence. The case study highlights the efficacy of the ACT model in addressing psychological barriers associated with injury recovery, underscoring the significance of MI in pain management and the improvement of sleep-related concerns.

To examine the potential effect of an adapted ACT intervention on individuals’ adherence to rehabilitation protocols and their overall psychological well-being, Mahoney and Hanrahan (2011) implemented a one-on-one educational course for four athletes who had undergone knee joint reconstruction surgery and were expected to return to sports within one to four months. The course was divided into four parts, conducted weekly, and covered themes of cognitive defusion, mindfulness, acceptance, and values. After the fourth intervention session, researchers conducted semi-structured interviews to explore participants’ perspectives on the role of ACT intervention in psychological adaptation. The interviews revealed that all participants found the skills taught in the ACT course – particularly mindfulness – to be beneficial in accepting negative emotions such as depression, boredom, and anxiety, and in promoting compliance with rehabilitation behaviours.

Shortway et al. (2018) drew inspiration from ACT to design the “Return to ACTion” protocol, which aimed to cultivate mindfulness, acceptance, and psychological flexibility among injured athletes, and to improve their adherence to rehabilitation behaviours. This protocol spanned 12 weeks and consisted of four modules. Each module included instructional segments, experiential exercises, and discussion points. Notably, the second module focused on mindfulness teaching and practice related to physical sensations, featuring an eyes-closed exercise called “Ice Bag”. In this practice, participants applied an ice bag to their wrist or arm, focusing attention on the sensations and noting any arising thoughts, memories, images, or feelings. Module 3 incorporated a popular mindfulness exercise known as “Leaves on a Stream”. Following the design of the protocol, researchers conducted an exploratory investigation to collect qualitative data, such as researcher observation logs and interactions with sports coaches, and validated the feasibility of their designed intervention protocol.

In addition to the “Return to ACTion” protocol, Moesch (2023) designed the MABI protocol aimed at promoting the recovery of acutely injured athletes. This protocol consisted of two parts: an 8-week online intervention program and four individual sessions. In the online program, injured athletes participated in guided mindfulness practices through the “Mindfulness for Stress” thematic course (Mindfulnesscenter, 2024). This course included a total of 960 minutes of mindfulness practice, with each session lasting 10 minutes and occurring six times per week. Each week offered different MI thematic courses covering topics such as “Taking a Break and Getting Started”, “Feeling Your Body”, “Enhancing Your Concentration”, “Calming Your Thinking”, “Stretching Your Limits”, “Accepting Things as They Are”, “Just Sitting”, and “Stop, Observe, Accept, Let Go or Respond”. These courses encompassed various types of mindfulness practices, including body scans, breathing anchors, mindfulness yoga, and sitting mindfulness. The individual sessions were tailored to meet the specific needs and psychological conditions of the injured athletes. These four individual sessions were scheduled at the start of the intervention, two weeks post-intervention, five weeks post-intervention, and eight weeks post-intervention. The content of these sessions included (1) mindfulness, attention control, and defusion, (2) body awareness and life values, (3) committed action and acceptance, and (4) open awareness and handling difficult situations.

Moesch et al. (2020) utilized a single-case design with multiple staggered and non-concurrent baselines to investigate whether MABI could enhance mindfulness levels (including acting with awareness and nonreactivity), acceptance, and psychological well-being, as well as reduce symptoms of anxiety and depression. The study randomly assigned participants to one of three groups, with baseline periods lasting two, three, or four weeks. The 8-week intervention comprised two components. The first was a web-based program, “Mindfulness Basics”, which required participants to undertake 10 minutes of formal mindfulness practice, such as breathing mindfulness and body scan, twice daily for six days each week, totalling 960 minutes. The second component consisted of four individual 1-hour sessions held in weeks one, two, five, and eight. The initial session was conducted in person at a convenient location, and the remaining three sessions were delivered online and synchronized with the web-based program. The sessions aimed to integrate the skills and experiences from the initial part of the intervention with injury recovery and to include content on life values and committed action. These sessions were facilitated by a co-author of the study, a sport psychologist with expertise in sport psychology, cognitive-behavioural therapy, and mindfulness. Participants completed repeated measures of questionnaires during the baseline (three to five times per week), intervention (four times every two weeks), post-intervention (twice every two weeks), and follow-up assessments 10 weeks post-intervention. The findings indicated that the intervention positively influenced injured athletes’ nonreactive mindfulness, acceptance levels, and psychological well-being, but did not significantly impact anxiety and depression symptoms.

Mohammed et al. (2018) conducted a study to determine if mindfulness-based stress reduction (MBSR) could decrease pain perception, increase pain tolerance and mindfulness levels, and enhance positive emotions during the rehabilitation of injured athletes. They recruited 20 athletes who had been inactive in sports for over three months due to sports injuries. These athletes were randomly assigned to either an intervention group (*n* = 10) or a control group (*n* = 10). The intervention group participated in an 8-week MBSR course, which included practices such as mindful breathing, body scan mindfulness, and sitting mindfulness, with weekly 90-minute sessions. Additionally, they were instructed to perform daily informal mindfulness practices at home, following guidelines from a provided compact disc, dedicating about 20 to 30 minutes to each session. The control group, in contrast, did not receive any specific intervention during their recovery period. The study’s results indicated that while positive emotions improved in both groups, the intervention group showed significant enhancements in both pain tolerance and mindfulness levels.

In regards to the applicability of MI among injured athletes, our synthesis of three included experimental studies revealed considerable variability in intervention dosage (ranging from 720 to 1200 minutes), intervention timing (spanning from 1 to 6 months post-injury), and selection of MI implementers (including sport psychologists with extensive MI experience and professionals trained in relevant mindfulness courses) (Mahoney & Hanrahan, 2011; Moesch et al., 2020; Mohammed et al., 2018). Nevertheless, it was also observed that the applicability of MI encompassed a broad spectrum of sports types (such as skiing, football, sailing, tennis, kickboxing, bodybuilding, running, basketball, and cycling) and various levels of athletic proficiency, from elite athletes to amateur practitioners.

## Discussion

This study aims to review the role of MI in the psychological adaptation of athletes following injuries. While limited studies were identified through a mixed-method search and reference mining from topic-related systematic reviews, our evidence synthesis from the included articles reveals the positive effects of MI on the psychological adaptation of injured athletes. Based on our synthesis, we discuss the utilization of MI after sports injuries from six aspects: (1) dosage (including upper and minimum limits), (2) timing, (3) methods (including online vs. offline and group vs. individual), (4) implementers, (5) intervention types, and (6) four limitations of existing MI approaches, such as unverified efficacy, restricted applicability to certain types of sports injuries, minimal impact on physiological recovery outcomes, and the potential for adverse effects.

First, the three experimental studies included in this paper show significant variations in intervention dosage (Mahoney & Hanrahan, 2011; Moesch et al., 2020; Mohammed et al., 2018). In Moesch et al. (2020) study, participants underwent a total of 1200 minutes of MABI, which included 96 online sessions (960 minutes) and four offline sessions (240 minutes). Moesch (2023) subsequently recommended a minimal dosage of 1200 minutes for MABI targeting the psychological rehabilitation of athletes post-sports injuries. Meanwhile, Mohammed et al. (2018) provided injured athletes with eight sessions of MBSR, totalling 720 minutes, conducted over eight weeks. Mahoney and Hanrahan (2011) implemented a 4-week ACT intervention with four sessions; however, the total intervention time for these sessions and the duration of MI alone, was not explicitly stated. Although these three studies conducted varying MI dosages, all showed promising results. Nevertheless, the MI dosage of four sessions within four weeks in Mahoney and Hanrahan’s (2011) study fell below the recommended minimum MI dosage of eight weeks for athletes (Basso et al., 2019). Considering this discrepancy in minimum intervention dosage, future research could delve deeper into determining the optimal minimum MI dosage for the psychological adaptation of injured athletes.

Additionally, while research has identified a proportional relationship between the dosage of MI and their effectiveness, the exploration of the upper limit dosage of MI after sports injuries remains undetermined (Creswell, 2017). Moreover, determining the upper limit dosage – which includes the frequency of dose interventions and the duration of a single intervention – may pose potential challenges in terms of adherence to rehabilitation practices and the operability of the intervention (Moesch, 2023; Mohammed et al., 2018). Therefore, future research could benefit from not only investigating the minimum MI dosage discussed above but also exploring the upper limit dosage for MI concerning the psychological adaptation of injured athletes.

Second, the three experimental studies conducted MI at different points during the rehabilitation process of injured athletes (Mahoney & Hanrahan, 2011, Moesch et al., 2020; Mohammed et al., 2018). While all these studies demonstrated a positive impact of MI on psychological adaptation during recovery, Moesch (2023) suggests that practitioners implement MI early in the athlete’s recovery process, as athletes often exhibit the most intense emotional reactions immediately after an injury. However, Clement et al. (2021) found that immediate MI did not alleviate negative psychological health consequences in patients with traumatic injuries from the general population. They attribute this contradictory finding to factors such as individual differences among participants, environmental influences, the Hawthorne effect, insufficient intervention dosage, and inadequate participant compliance with mindfulness practice. Considering these potential influencing factors on MI outcomes, future experimental designs may need to account for and control these confounding variables and identify the optimal timing for implementing MI (e.g., immediately after injury, post-surgery, during the recovery period, or during the return-to-sport process).

Third, all studies included in this research mentioned offline MI, but three of them specifically highlighted a promising implementation method: online MI (Moesch, 2023; Moesch et al., 2020; Mohammed et al., 2018). Online MI has shown promising potential in influencing athletes’ sports performance (Forbes & Johnson, 2022). It can be implemented through web-based programs such as mobile applications (e.g., Headspace, Calm, and Smiling Mind) and courses available on online platforms. Compared to offline MI, online strategies offer greater flexibility and feasibility (Anderson et al., 2021), eliminating concerns about potential disruptions to the experimental process (Moesch et al., 2020; Mohammed et al., 2018). Particularly, this method provides easily accessible psychological interventions for athletes who may face mobility challenges due to sports-related injuries (Moesch, 2023). However, the potential differences in effectiveness between online and offline MI warrant further exploration, especially considering the importance of social support interactions during offline group MI for injured athletes (Bennett & Lindsay, 2016).

Fourth, among the three experimental studies, only two mentioned the intervention implementers. These were either sport psychologists with extensive experience in MI (Moesch et al., 2020) or professionals who had received relevant mindfulness course training (Mahoney & Hanrahan, 2011). The qualifications and experience of the implementers are crucial to the experimental outcomes of MI (Ruijgrok-Lupton et al., 2018). Therefore, MI implementers should be qualified mindfulness practitioners with formal training in relevant mindfulness courses (Myall et al., 2023). Furthermore, Moesch (2023) recommends that MI implementers should also have a robust background in sport psychology to better address the various psychological challenges faced by injured athletes. Importantly, all this information should be thoroughly documented in their research to enhance the transparency and reproducibility of the experimental studies.

Fifth, while these three experimental studies applied MI to injured athletes, they utilized different experimental designs. For example, Mohammed et al. (2018) solely employed MBSR, whereas the other two studies integrated MI into other intervention modalities such as ACT and MABI for joint intervention on injured athletes (Mahoney & Hanrahan, 2011; Moesch et al., 2020). Notably, these diverse mindfulness approaches consistently demonstrated positive effects on the psychological adaptation of athletes following injuries. However, considering the wide range of mindfulness methods, beyond the types identified in this paper, other widely recognized approaches in sport psychology include mindfulness, acceptance and commitment, mindfulness meditation training in sports, and mindful sport performance enhancement (Gledhill & Forsdyke, 2021). Each distinct mindfulness type may yield varying effects on athletes (Matko & Sedlmeier, 2019). Therefore, future experimental research on the psychological adaptation of injured athletes could explore contrasting these different types of MI, which would aid in determining the most effective MI approach for enhancing psychological adaptation after sports injuries. Given that MI is often integrated into other psychological interventions, such as CBT, MBCT, and ACT (Bredero et al., 2023; Herbert et al., 2022; Interian et al., 2023), future researchers exploring the efficacy differences of integrated intervention therapies by incorporating MI into other psychological treatment regimens would be a promising endeavour.

Lastly, through the evidence synthesis of the review study, we identified four limitations of existing MI designs for psychological adaptation after sports injuries. Firstly, while the Return to ACTion intervention protocol designed by Shortway et al. (2018) received positive evaluations from sports coaches, its acceptance, feasibility, and effectiveness remain unclear due to a lack of practical testing by injured athletes. Thus, its real-world application effects are yet to be determined. Secondly, MABI designed and applied by Moesch et al. (2020) and Moesch (2023), specifically tailored for athletes experiencing acute injuries, may not be suitable for athletes with overuse injuries, as they may face different psychological challenges, leaving a research gap for designing MI suitable for these athletes. Thirdly, despite the positive impact of MI on the psychological adaptation of injured athletes during the recovery period, research by Sole et al. (2021) suggests that MI may not be related to the physical recovery time from injuries. In other words, while MI can enhance psychological adaptation and improve the likelihood and outcomes of returning to sports (Anderson et al., 2021; Owusu-Ansah et al., 2023), it may not necessarily shorten the time it takes for injured athletes to return to sport. Fourthly, while the experimental studies included in this research consistently indicate the positive impact of MI on psychological adaptation, they have not explored the potential adverse effects of MI on mental health. For instance, a recent systematic review incorporating 83 articles and 6,703 participants found that 8.3% of participants experienced adverse events (e.g., anxiety, depression, cognitive abnormalities, and even suicidal behaviour) after engaging in mindfulness practices (Farias et al., 2020). Given the heightened vulnerability to psychological challenges among injured athletes, future research on psychological adaptation interventions for athletes recovering from injuries should investigate the potential adverse psychological effects of MI on injured athletes. These research endeavours can better assist sport psychologists in designing MI that facilitates psychological adaptation after sports injuries.

### Study limitations and implications

While this article conducted separate data extraction and summarization for the included experimental studies, it exclusively assessed the methodological quality of empirical articles when evaluating the overall quality of the included studies. As a result, the unclear quality of the one non-empirical article could impact the overall results of the evidence synthesis. Another limitation of this study is its exclusive focus on research composed in English, thereby excluding papers written in other languages. This choice may have resulted in a relatively limited pool of included results, potentially affecting the robustness of the conclusions. Nevertheless, due to the comprehensive search and detailed review undertaken on MI in the realm of psychological adaptation following sports injuries, and with both manual and electronic database searches yielding the same set of included results, the authors of this study express confidence in providing a thorough summary of the design and application of MI in the field of psychological adaptation after injuries.

Considering the inherent limitations in the existing body of research in this domain, and in light of the research gaps identified in this study, future researchers are encouraged to conduct more in-depth and comprehensive explorations of the role of mindfulness practices in fostering psychological adaptation after sports injuries. Furthermore, for practitioners, this study represents the inaugural systematic review examining the relationship between psychological adaptation in injured athletes and mindfulness practices. Therefore, the consolidated research findings provide practitioners with reliable insights into the dosage, timing, methods, types, and limitations of existing MI when applied to injured athletes. This contributes to a more nuanced understanding and application of MI in this specific domain.

## Conclusion

In conclusion, this systematic review highlights the positive impact of MI on the psychological adaptation of injured athletes, emphasizing its potential as a valuable tool in rehabilitation processes. The synthesized evidence reveals that diverse MI approaches contribute to mitigating anxiety and depression, enhancing pain tolerance, and improving mindfulness levels and overall psychological well-being among athletes recovering from sports injuries. However, future research should address the identified limitations, including exploring optimal dosage, timing, and potential adverse effects of MI. Additionally, the variations in intervention types and implementers underscore the need for standardized protocols. Despite these considerations, the findings of this review offer valuable insights for sport psychologists and practitioners seeking effective interventions to support the psychological adaptation of injured athletes during their recovery journey.

## Data Availability

The data that support the findings of this study are available from the corresponding author, [N, Y], upon reasonable request.
